# Healing of Chronic Wounds with Platelet-Derived Growth Factors from Single Donor Platelet-Rich Plasma following One Freeze-Thaw Cycle. A Cross-Sectional Study

**DOI:** 10.3390/jcm10245762

**Published:** 2021-12-09

**Authors:** Vidán-Estévez Julia, Sánchez-Herráez Sergio, Escalante-Barrigón Fernando, Seco-Calvo Jesús

**Affiliations:** 1 Servicio de Transfusión, Complejo Asistencial Universitario de León (CAULE), 24008 León, Spain; jvidan@saludcastillayleon.es; 2 Cooperative Working Group on Lymphomas and Lymphoproliferative Disorders of the Castilla y León Society of Hematology and Hemotherapy (SCLHH), Department of Haematology, 47007 Valladolid, Spain; 3 Servicio de Cirugía y Traumatología Ortopédica, Complejo Asistencial Universitario de León (CAULE), 24008 León, Spain; herraezsergios@yahoo.es; 4 Servicio de Hematología y Hemoterapia, Complejo Asistencial Universitario de León (CAULE), 47007 León, Spain; fescalanteb@yahoo.es; 5 Institute of Biomedicine (IBIOMED), University of León, 24071 León, Spain; 6 Visiting Professor and Researcher of Department of Physiology, University of the Basque Country, 48940 Leioa, Spain

**Keywords:** al-PRP, chronic, non-healing wounds, platelet-derived growth factor (PDGF)

## Abstract

Chronic non-healing wounds (CNHWs) may be associated with trauma or idiopathic in nature and are difficult to treat. Our objective was to assess the use of platelet-derived growth factor (PDGF) from single-donor platelets (al-PRP), using one freeze-thaw cycle, for treating CNHWs. We conducted a cross-sectional study. A total of 23 CNHWs being treated with al-PRP. The al-PRP treatment can be considered successful in well over half (*n* = 13, 56.5%) of the wounds. We found that all the wounds treated for up to 7 weeks showed partial or complete healing, while those treated for between 8 and 12 weeks did not show healing, healing again being successful in cases in which treatment was extended to more than 13 weeks (85.7%). Using chi-square tests, this relationship was found to be highly significant (*p* < 0.001, chi^2^ = 19.51; *p* value = 0.00006). Notably, Cramer’s V coefficient was very high (0.921), indicating that the effect size of PRP treatment duration on healing is very large (84.8%). We could suggest that the use of al-PRP in the healing of CNHWs is a promising approach. Further studies with larger sample sizes and long follow-ups are needed to obtain multivariate models to explain which factors favour the healing of ulcers treated with PRP

## 1. Introduction

Chronic non-healing wounds (CNHWs) may be associated with trauma or idiopathic in nature and are difficult to treat [1,2]. Their worldwide prevalence is estimated to be as high as 0.5–1% and they are known to be associated with high morbidity and mortality, impairing patients’ mobility and quality of life quality of life, causing ill-heath related absence from work (with some 6 million working days lost per year in the USA) and generating high healthcare costs (over 5 billion dollars per year in the USA) [3,4,5,6]. These figures underline the need to explore alternative approaches [7] to accelerate wound healing and thereby both improve patient quality of life and decrease costs.

The treatment of CNHW is recognised to be a major challenge [8], the response to current approaches, including debridement and offloading, tending to be disappointing. Indeed, these wounds are associated with high risks of infection, amputation and even death. Successful wound healing is a well-orchestrated biological and molecular process involving numerous complex events related to cell migration and proliferation as well as extracellular matrix deposition and remodelling [9] Seeking to optimise conditions for these events, several novel approaches have been proposed for the treatment of these wounds, including the use of platelet-rich plasma (PRP).

Widely used in the fields of tissue engineering and regenerative medicine, the potential benefits of PRP include a positive effect on migration, proliferation and angiogenesis in in vitro as well as animal and human-based studies [9,10,11,12]. It has already been applied in medical fields from traumatology to ophthalmology and dentistry [13,14,15,16,17,18,19]. By definition, PRP contains concentrated platelets; it is usually prepared from whole blood by centrifugation, and while the focus has initially been on autologous platelet-rich plasma (au-PRP), there is growing interest in allogeneic (al-PRP) platelet-rich plasma.

Specifically, au-PRP is recognised as an adjuvant therapy that is effective in promoting the healing of skin wounds in animals [20,21], but also as a feasible and safe treatment for chronic leg ulcers and, in particular, diabetic lower extremity ulcers (DLEUs) [22,23,24,25]. In the context of severely-infected diabetic foot ulcers, basic and clinical research suggest that au-PRP helps stimulate soft tissue regeneration and also has antibacterial properties [26,27]. Nonetheless, there are limitations to the application of au-PRP in patients with DLEUs, ranging from the severe complications and comorbidities that are common in such patients to a lack of reproducibility in the results of treatment with this product, related to the lack of a standardised protocol for preparing au-PRP [28].

This has motivated us to explore the reliability and safety of the effects of al-PRP. There has been relatively little research on this type of PRP beyond a few pilot and case studies in which it was found to have little immunogenicity and great healing efficacy in promoting tissue regeneration [29,30,31]. Specifically, to our knowledge, few data have been published on the use of al-PRP for CNHWs in general [32] or hard-to-heal DLEUs [33], in particular or concerning the safety and efficacy of al-PRP compared to those of au-PRP.

On the other hand, given the need for new approaches are needed to strengthen the treatment of CNHWs, we had started to explore of the use of al-PRP in some patients. Therefore, the objective of this study was to assess the use of platelet-derived growth factor (PDGF) from single-donor platelets (i.e., al-PRP), using one freeze-thaw cycle, for treating CNHWs. Our hypothesis was that al-PRP prepared in this way is both effective and safe for the treatment of this common clinical problem.

## 2. Materials and Methods

### 2.1. Study Design and Participants

We conducted a cross-sectional study and report it here in accordance with the Strengthening the Reporting of Observational Studies in Epidemiology (STROBE) statement (https://www.strobe-statement.org/) (accessed date: 20 October 2021). Participants were recruited from among patients with CNHWs treated at the Transfusion Unit of the Complejo Asistencial Universitario de León (CAULE), between January 2016 and February 2021, with PDGF in PRP obtained by single-donor apheresis. In all cases, the treatment was carried out using donor platelets, as autologous donations were deemed inappropriate, due to patients’ having advanced age or active cancer or chronic myeloproliferative syndromes, and in particular poor venous access.

#### Inclusion and Exclusion Criteria

Inclusion criteria: Patients were required to have CNHWs with a poor response to other treatments, referred from various different specialities including haematology, vascular surgery, and traumatology and orthopaedic surgery, and as well as haematology patients with CNHWs secondary to the use of hydroxyurea (Hydrea^®^); and poor venous access, making them candidates for al-PRP rather than au-PRP; as well as be 18 years old or more and have given written informed consent.

Exclusion criteria: Patients were excluded if they had a poor nutritional status (severe nutritional deficiency, subclinical status or body composition indicating malnutrition); a concomitant infectious condition (positive serological results for hepatitis B surface antigen, anti-hepatitis C virus, anti-HIV, or syphilis, as well as polymerase chain reaction for hepatitis B and C viruses and HIV, or infected skin ulcers); advanced age, over 90 years old; or inability to perform their own wound care or have it performed by a caregiver every day for an extended period (typically 2 to 4 months) until the ulcer healed (due to difficulty reaching the site of their ulcers, associated with limited mobility or functioning and living alone).

### 2.2. Procedure

#### 2.2.1. Plateletpheresis

Donor whole blood is fractionated by centrifugation using a Spectra Optia Apheresis System (Terumo BCT, Lakewood, CO, USA). Blood is collected from a large vein, generally in the antecubital area, and transferred, through a sterile device, to a centrifuge spinning at 2400–2800 rpm, depending on the hematocrit value, platelet count and target performance for the procedure. The blood cell separator identifies the different components, based on their weight and density, platelets being transferred to a collection bag, while the other components return to the donor’s bloodstream.

The procedure takes around 90 min and donors must meet the eligibility requirements for apheresis donation: good general health with no abnormal lab results, a bodyweight > 50 kg, age > 18 and <65 years, good venous access in the cubital area in both arms, and no history of bloodborne illnesses (hepatitis B or C, AIDS, syphilis) or abnormal bleeding, as well as not having taken salicylic acid or derivatives thereof or other anti-inflammatory drugs in the previous 5 days, and having a platelet count >150 × 10^3^/uL.

Having completed the apheresis procedure, the PRP is labeled with the unit number, type of product, ABO blood group, and Rh (D), date of collection, expiry date, lab results, type of anticoagulant used, and storage and administration conditions. Until further processing (aliquoting), this PRP is stored under constant stirring at 22 °C for up to 7 days. Given the nature of the process, the use of allogeneic ABO-identical platelets obtained by transfusion services, which ensure safety standards in pre-transfusion practices, is an approach employed in regenerative medicine that facilitates the use of platelets that retain the ability to release growth factors (specifically PDGF) and other bioactive compounds.

#### 2.2.2. Aliquoting

After selecting a platelet apheresis unit, the unit number to be aliquoted for the patient is registered in our e-Delphyn^®^ database ensuring the traceability of the product. The apheresis product is transferred to a laminar flow cabinet (Telstar^®^) in the cryopreservation room for aliquoting. Working under a laminar flow creates a particle-free environment, air being taken in through a filtration system and reaching the work area as a unidirectional stream. The cabinet is closed on the sides and is kept under constant positive pressure to avoid inflow of contaminated air from the environment (HEPA filter). Additionally, microbiological cultures of cabinet surfaces (worktop, as well as the sides and top) are taken monthly, confirming that the working area is sterile before every aliquoting procedure. Depending on the size of the lesion to be treated, the entire apheresis product is aliquoted into 2.5- or 5-mL syringes. Subsequently, the aliquots are labelled with the patient´s name, and the quantity of product, preparation and expiry dates and stored in bags of seven aliquots, corresponding to the amount to be delivered to the patient each week.

#### 2.2.3. Cryopreservation

All the clearly labelled aliquots are stored in an ultra-low freezer at −80 °C (Thermo Scientific, Hosingen, Luxembourg) in the CAULE Transfusion Services, ensuring the stability of the product for up to 6 months.

#### 2.2.4. Application Procedure

Each week, the patients collected the daily doses (seven aliquots) from the Transfusion Service and stored them in their home freezer. Each day, he thawed the daily dose in a water bath and, having cleaned the wound, used the syringe to apply the product across the entire area to be treated. He was instructed to then rest in a supine decubitus position for at least 6 h, to minimize his movement and loss of the product, thereby ensuring the wound treated had a long exposure to the PRP. Once the al-PRP is thawed, it is placed directly on the wound, left uncovered for half an hour. Subsequently, the wound and product were covered with a sterile gauze until the following day.

The study was carried out through the CAULE Transfusion Service by a haematologist, who was in charge of supervising the treatment. Qualified nurses from this service carried out the relevant nursing procedures, in particular, wound cleaning and dressing changes, strictly following the protocol established by CAULE for nursing care associated with the use of PRP for local treatments.

### 2.3. Statistical Procedure

Qualitative data were expressed as frequencies and percentages. Contingency tables were used for comparing these variables two by two. Quantitative data were expressed as mean, median, standard deviation and total range. The non-parametric Mann-Whitney U test was used for comparing the means of numerical variables.

The chi-square test of independence for categorical variables was used to assess whether there was a relationship between variables. If there is such a relationship, it can be inferred that there are significant differences in the response variable between the categories of the factors, using the values of the adjusted standardised residuals (similar to Z-scores for normal distributions, for which residuals ≥ 2 is considered to indicate significance).

To express the magnitude of the differences between groups, effect sizes were calculated and expressed as R2 (on a scale: 0–1) enabling comparisons between different types of data describing the variables and different types of statistical tests. Specifically, we used we used Cohen’s d when comparing means [34,35] and Cramer’s V coefficient for categorical variables and the chi-square tests.

In all the inferential statistical tests, *p* values < 0.05 were considered to indicate that an association was significant and a *p* < 0.01 that it was highly significant.

The statistical analysis was carried out using IBM-SPSS Statistics^®^ version 25 (IBM Corp. Released 2017. IBM SPSS Statistics^®^ v 25.0 for Windows; Armonk, NY, USA), except for effect size calculations, for which we used G*Power freeware version 3.1.9.7 (available at: http://es.softoware.org/apps/download-g-power-161725-for-windows-7-os.html) (accessed date: 7 January, 2016).

### 2.4. Ethical Considerations and Participant Involvement

All procedures were carried out in accordance with the principles of the Declaration of Helsinki (2013, revised 5 May 2015) and in compliance with the ethical regulations and Spanish law on the protection of personal data (15/1999) and biomedical research in humans (14/2007) and Organic Law 3/2018 of December 5th on the protection of personal data and guarantee of digital rights, as well as the General Data Protection Regulation (EU) 2016/679. Specifically, in accordance with the Organic law 15/1999, we set up an encrypted data collection form. In this regard, the University of Leon has a data protection officer, in charge of reporting, assessing and supervising adherence to the legal obligations regarding the handling of data (identification of data protection officer: Start Up, S.L. contact email: dpd.unileon@seguridadinformacion.com).

The study was approved by the Ethics and Clinical Research Committee of the University of Leon (n° COD: ETICA-ULE-004-201) and all patients gave written informed consent before inclusion.

In addition, the Spanish Agency of Medicines and Medical Devices (AEMPS) considers PRP to be a medicinal product for human use that can be used under Article 5 of Directive 2001/83/EC of November 6 and the provisions transposing the Directive into Spanish law. The autologous plasma and its fractions, components and derivatives have been used in accordance with the provisions in the Resolution of 23 May 2013 and the report on the use of these products published on the AEMPS website (available at: https://www.aemps.gob.es/medicamentosUsoHumano/medSituacionesEspeciales/faqs-terapeutico-plasma-autologo.htm) (accessed date: 3 January 2016).

Further, this study was conducted in strict compliance with all requirements of the current legislation, as well as the standards in terms of quality, efficacy, traceability, information and pharmacovigilance stated in the AEMPS Report on the use of PRP (Report/V1/23052013) (available at: https://www.aemps.gob.es/medicamentosUsoHumano/medSituacionesEspeciales/docs/PRP-AEMPS-DEF-mayo13.pdf) (accessed date: 3 January 2016).

### 2.5. Patient and Public Involvement

Patients were not, however, invited to comment on the study design, consulted to develop patient-relevant outcomes, or interpret the results, or invited to contribute to the editing of this paper.

## 3. Results

**Figure 1:**
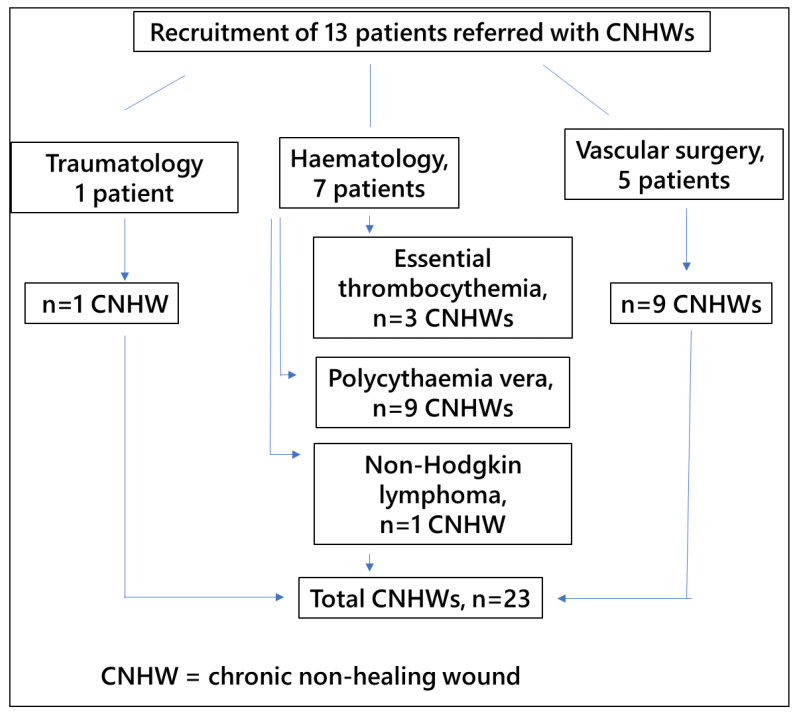
Flow chart of patients included in the study.

Among patients recruited by the Transfusion Unit to start this treatment with al-PRP, seven were referred from the Haematology Unit, six with a haematological diagnosis of chronic myeloproliferative disorders such as essential thrombocythemia (ET) or polycythaemia vera (PV) treated with hydroxyurea, and one with a diagnosis of non-Hodgkin lymphoma. These patients with haematological disorders had a total of 13 wounds to be treated, some individuals having more than 1 wound. Further, five patients were referred from vascular surgery, with a total of nine wounds, and one patient from traumatology and orthopaedic surgery, with a single wound (Figure 1).

In relation to the demographic characteristics of the sample, the age range was 53–89 years old, with a median of 75.3 ± 10.1 (95% CI: 70.9–79.7) and a median of 77 years old; most of the participants (39.1%) being aged between 75 and 79. In terms of sex, 69.6% of the patients were men and 30.4% were women. The mean age of the men was significantly lower than that of the women (78.2 vs. 81.6) (*p* < 0.05, Mann-Whitney U test: Z-score = 2.02; *p*-value = 0.029).

Regarding blood group, over half of participants were group O, and the great majority were Rh+. In line with this, the most common blood groups were O+ (43.5%), followed by A+ (34.8%). All patients tested negative for irregular antibodies and obtained negative serological results for the diseases analysed. Table 1 summarises the blood test results.

Characterising the wounds, most were found to be on legs (65.2%) or areas associated with the lower extremities (e.g., the ankle or foot, 30.4%). They ranged from 0.5 to 8 cm in diameter, with a mean of 3.1 cm. Regarding severity, 21.7% of the wounds were classified as severe and 34.8% as moderate-to-severe; that is, considering both categories, more than half (56.5%) of the wounds could be considered major.

Regarding history of wound care, most patients in the sample (43.5%) had received local wound care at hospital and the second most common approach was wound care at home (30.4%), but as many as a quarter (26.1%) had undergone surgery. The mean duration of previous treatment was more than 3 months, ranging from 1 to 10 months; less than half (47.8%) had been treated for less than 3 months, 21% for 3 months, and nearly a third (30.4%) of the sample for more than 3 months.

The duration of treatment with PRP ranged from as short as 4 to as long as 40 weeks, with a mean of 13 weeks. We found that all the wounds treated for up to 7 weeks showed partial or complete healing (100%; 7 wounds), while those treated for between 8 and 12 weeks did not show healing (100%; 9), healing again being successful in cases in which treatment was extended to more than 13 weeks (85.7%; 6 out of 7). Using chi-square tests, this relationship was found to be highly significant (*p* < 0.001, chi2 = 19.51; *p* value = 0.00006). Notably, Cramer’s V coefficient was very high (0.921), indicating that the effect size of PRP treatment duration on healing is very large (84.8%).

**Figure 2:**
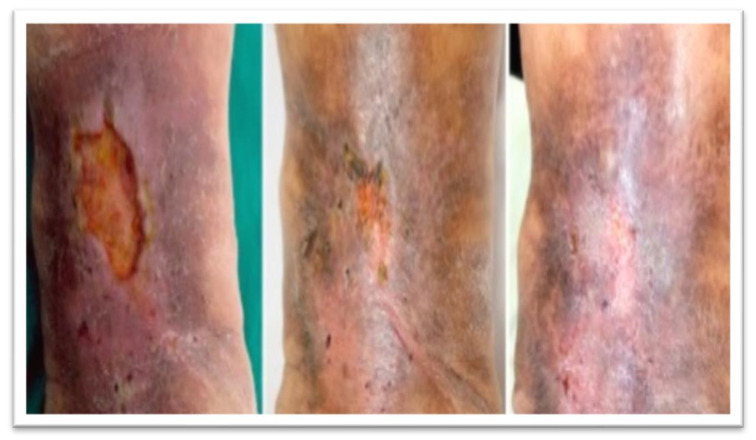
Example of wound healed completely (left, week 0; middle, week 7; right, week 12).

**Figure 3:**
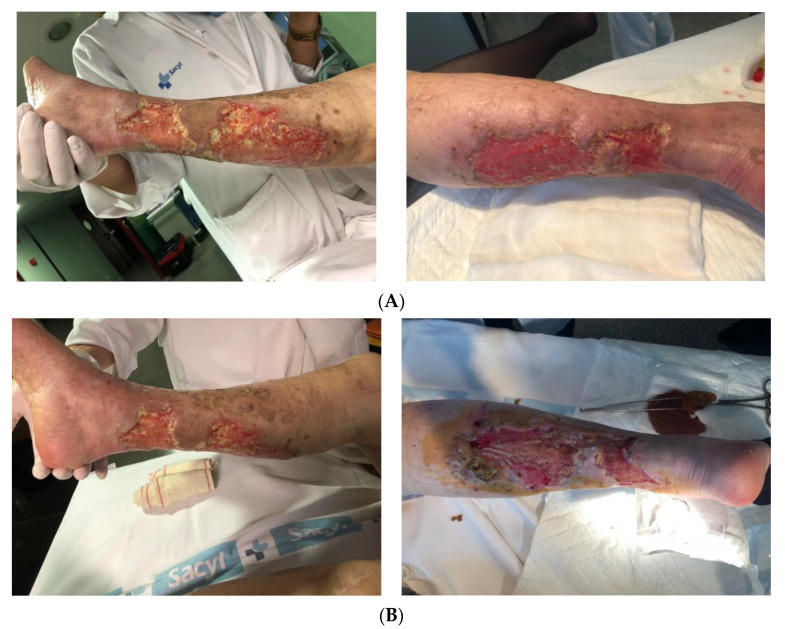

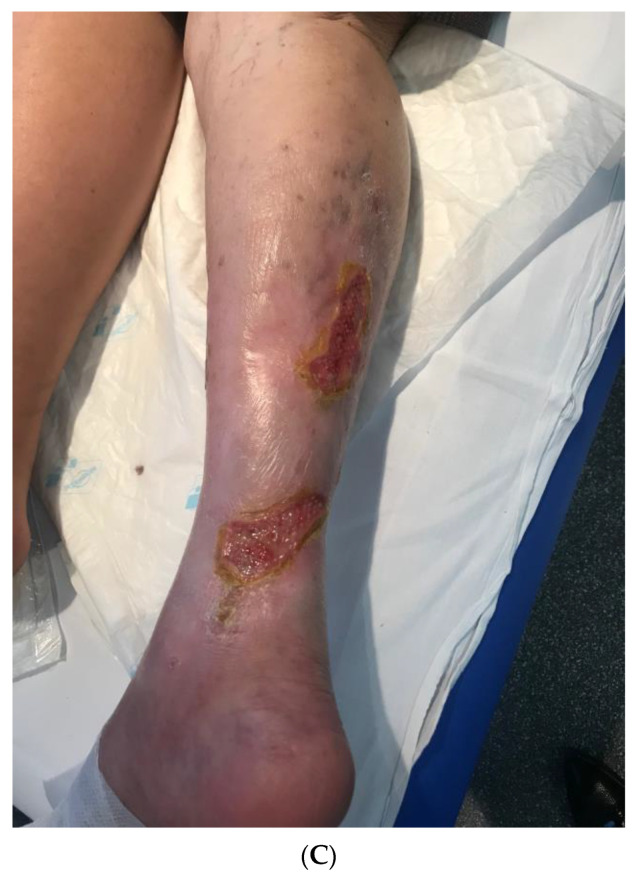
Example of wound healed partially. (**A**). Week 0. Example of wound healed partially. (**B)** Week 4. Example of wound healed partially. (**C**) Week 16.

Assessing the extent of healing, 34.8% of wounds healed completely (Figure 2), no further treatment being required, while 21.7% showed partial healing (Figure 3), defined as a 50% reduction in wound size. Therefore, we could consider that the treatment was successful in 56.5% of wounds, that is, all the cases in which some level of healing was observed. Among the other wounds, infections developed in 34.8% of cases.

### 3.1. Healing-Related Factors

The mean age of patients in whom we observed healing was nearly 10 years younger than that of patients in whom PRP treatment was not successful. Though this difference did not reach significance (0.05 < *p* < 0.10), it corresponded to a large effect size (nearly 20%). Categorising patient age by decades (Table 2), we found that healing was achieved in all patients under 70 years old, but also that while the treatment success was lower in 70- to 79-year-olds, there was an increase in the healing rate among patients over 80 years old. With this analysis, the level of significance remained similar (*p* < 0.10) and the effect size was even larger (nearly 25%).

Comparing between the sexes (Table 2), the rate of healing was higher among men (68.8% vs. 28.6% in women), but not significantly (*p* < 0.10), though the effect size was large (almost 14%).

### 3.2. Medical History

Rates of healing were higher in those without comorbidities, and among those with relevant underlying conditions, in those with DM and cardiovascular diseases, but the differences did not reach significance, likely due to the relatively small sample size (Table 3). Notably, considering *p* values and effect sizes, neither having high blood pressure nor having DM emerged as factors associated with healing.

On the other hand, the rate of healing seemed to be lower among patients who had been treated with hydroxyurea, though the results did not reach significance; nonetheless, the medium effect size (11.2%) may suggest a relationship that could be explained by a negative effect of hydroxyurea.

### 3.3. Blood Test Result-Related Factors

Healing seemed to be easier in patients with group A or B blood and those who were Rh+, but the differences were not statistically significant, despite medium effect sizes which might suggest a relationship (Table 4). Further, healing was somewhat faster in patients with abnormal haemoglobin levels (high or low), but differences in means were non-significant and the effect sizes small, subgroups being too small for this finding to be reliable.

On the other hand, a statistically significant association was found for white blood cell count, considering both quantitative and categorical variables. Specifically, healing was associated with a lower white blood cell count (7.85 × 10^9^/L vs. 11.41 × 10^9^/L). This is confirmed by the higher healing rate among patients with normal levels (73.3% vs. 25% among those with elevated levels). No relationship was found between healing and platelet count, while for lymphocyte count, the differences did not reach significance but the effect size was large, suggesting wounds were more likely to heal in patients with lymphopaenia (83.3% vs. 47.1% among those with normal lymphocyte count).

Neither albumin nor quantitative cholesterol levels were associated healing. Interestingly, however, when categorised, there was a striking significance (*p* < 0.01) and a very large effect size (41%), indicating a lower level of healing among people with normal cholesterol levels (33.3% vs. 100% among those with abnormal levels, either high or low).

### 3.4. Wound-Related Factors

As 12 out of 13 patients had wounds in the same anatomical region, we were unable to explore the effect of wound site. Regarding wound size, differences did not reach significance and while the effect size might suggest a relationship, we doubt there is a real trend as healing was better in both small and large wounds (Table 5).

Mild and moderate wounds were more likely to heal than more severe wounds (100% and 62.5%, respectively, vs. ≤50%), as expected, but differences did not reach significance and the effect size was medium.

Analysing previous treatments, again differences did not reach significance, though there was a medium effect size suggesting that healing may be less common in patients with a history of surgical treatment, but healing was not related to the duration of previous treatments.

To summarize, with our relatively small sample size, two variables were found to be significantly associated (*p* < 0.05) with successful healing: an abnormal cholesterol level (high or low vs. normal) and normal white blood cell count. Additionally, there were four other variables for which associations did not reach significance, but which had effect sizes suggesting they may have some relationship with healing: age, sex, lack of comorbidities and lymphocyte count.

## 4. Discussion

To the best of our knowledge, this study is the first study of patients who have received PDGF from single donor PRP (al-PRP) processed with just one freeze-thaw cycle for treating their CNHWs. In this study, the al-PRP treatment can be considered successful in well over half (*n* = 13, 56.5%) of the wounds, in that it was associated with either complete (*n* = 8, 34.8%) or partial (*n* = 5, 21.7%) healing. Among the other wounds treated (*n* = 10, 43.6%), infections developed a third of cases (*n* = 8, 34.8%) and the treatment was discontinued in two (8.7%). While our group has gained experience in the management of PRP obtained from donors since January 2012 [36], and in particular has used ABO-identical platelets from single healthy donors with promising results [31], we have only reported our work regarding their use for skin ulcers and secondary mandibular osteonecrosis at scientific meetings [36]. We emphasize that this current work with new recruited patients continues our previous studies and also the results are now consistent and support the previous results obtained.

### 4.1. Difficult-to-Heal Wounds

Over half of the wounds treated in our study were in patients receiving treatment with hydroxyurea. In many patients, chronic lower extremity ulcers are a serious adverse effect of long-term treatment with hydroxyurea at doses of at least 1 g/day. These ulcers tend to develop in the malleolar region, and if allowed to persist, may become cancerous but usually heal spontaneously when hydroxyurea treatment is discontinued [37,38]. Indeed, to be successful, traditional wound care -involving debridement and various types of dressings- seems to require discontinuation of hydroxyurea. It is well known that hydroxyurea can inhibit the functions of ribonucleotide reductase, which in turn reduces the production of DNA in the cell and induces cell death. As well as inhibiting DNA synthesis, hydroxyurea has key antiangiogenic properties [37]. In patients for whom discontinuation is not appropriate, such as those who are unable to tolerate other drugs, there is a lack of effective strategies to achieve wound healing. Notably, in our sample, 41.7% (*n* = 5) of the hydroxyurea-related wounds healed partially or completely.

On the other hand, in our study, nearly a third of the wounds treated were in patients who had a diagnosis of DM. It is known that DLEUs are hard to heal, the difficulty being attributed to a loss of growth factors related to wound healing and an inflammatory imbalance in the bed of the wound [39,40]. In this context, various novel biological therapies seek to regulate and reverse mechanisms related to this imbalance [41,42]. Specifically, Hu et al. [43] in their systematic review and meta-analysis, including 431 patients with diabetic ulcers from 8 randomised controlled trials, found a higher rate of complete ulcer healing and greater reductions in ulcer size in patients treated with au-PRP. In our series, nearly three-quarters of the DLEUs (71.4%, *n* = 5) showed complete or partial healing.

### 4.2. Role of PRP in Wound Care

By promoting safe and natural healing, PRP is a promising alternative to the current standard of care for hard-to-heal wounds such as those of our patients, many having a history of DM and/or treatment with hydroxyurea and all a poor response to other treatments. This product is obtained from density gradient centrifugation of peripheral venous blood and contains a wide variety of growth factors, mesenchymal stem cells, fibroblasts and white blood cells [44]. Some studies have confirmed that it can promote wound healing, improve angiogenesis, inhibit the growth of various bacterial species and reduce postoperative pain [45,46]. There is growing evidence that PRP improves the healing of chronic wounds and the use of this product is increasing in the field of wound repair [47].

The strategy to promote wound healing using PRP/platelet gel is to prepare the product and apply it to sites of surgical interventions, injuries and non-healing wounds, as in the case of our patients, thereby delivering growth factors directly to the wound site [48]. The mechanisms of action are believed to involve molecular and cell induction of normal wound healing, similar to that triggered by platelet activation [49,50,51]. In the case of au-PRP, there is evidence that it releases growth factors and antibacterial peptides boosting antibacterial effects, favouring wound repair, and alleviating pain associated with CNHWs [26,27,48,52,53].

The function of both au- and al-PRP relies on platelet degranulation resulting in a high concentration of various growth factors, including fibroblast growth factor, transforming growth factor, insulin-like growth factor, vascular endothelial growth factor and platelet-derived growth factor [26,54], that play a key role in tissue repair and regeneration. Additionally, PRP has been found to have antibacterial properties. For example, in vitro, it was found to inhibit the growth of *Pseudomonas aeruginosa*, *Staphylococcus aureus* and *Streptococcus faecalis* for as long as 2 h of incubation, through the induction of C-C motif chemokine ligands 3 and 5 and C-X-C motif chemokine ligand 1 [55], and in a study in dogs, PRP activated by calcium chloride showed antimicrobial activity against infection by methicillin-resistant *Staphylococcus aureus* and this was associated with better re-epithelisation and granulation tissue formation improving the healing of the infected wounds [56].

In relation to clinical practice, PRP provides a biological antibacterial agent that does seem to be effective in the treatment of diabetic skin ulcers with severe multidrug-resistant bacterial infections [26,57,58,59]. Despite the potential antimicrobial activity of PRP, however, infections developed in two of our patients and their ulcers did not heal.

A pilot study [29] comparing the use of al-PRP with current best practice wound care failed to find significant differences in the reduction in chronic pressure ulcer volume between the groups. Nonetheless, the rate of proliferation of granulation tissue was significantly faster in al-PRP-treated patients than in controls during the first 2 weeks of treatment, suggesting that al-PRP may stimulate granulation tissue proliferation in the early stages of healing [29]. More recently, significantly faster healing has been observed with either al- or au-PRP, wounds healing in a mean of <60 days versus >85 days with conventional wound care [33].

Interestingly, in our study, rates of success varied over time, with wounds either healing relatively fast (within the first 7 weeks) or requiring extended treatment (of 13 weeks or more). On the one hand, this implies the need to underline to patients that it may take some time to obtain a positive response to this treatment. On the other, it implies a need for further longitudinal studies to help explain this pattern of response. Specifically, in our series, we did not find the clinical course of the ulcers to be related to DM, hypertension, or lymphocyte count, that is, these factors did not influence either the variable describing improvement in the wound or treatment duration. Just two factors were significantly related to successful healing, namely, an abnormal cholesterol level for which we have found no plausible explanation in the literature and normal white blood cell count, which might be expected (increases in white cells being a known response to infection and inflammation). Given the nature of the study, however, we cannot infer causality and both findings need confirmation with larger samples. Overall, there is a clear need for more data to determine factors that would help identify good candidates for al-PRP treatment and we consider this a very interesting area for future studies.

### 4.3. Use of al-PRP vs. au-PRP in the Treatment of Chronic Wounds

Systematic reviews and meta-analyses have found that au-PRP therapy improves the rate of complete and partial healing of hard-to-heal wounds compared to standard wound care [49,60]. So far, however, al-PRP has not been widely used in clinical applications, the main barrier being the risk of immune reaction and cross contamination. Nonetheless, no adverse reactions were noted in our patients, consistent with the findings of He et al. [33], who observed no evident local inflammation, allergies or other adverse reactions in patients treated with al-PRP, similar to observations in patients treated with au-PRP. We speculate the following potential explanations for these findings. First, the al-PRP was prepared with ABO and Rh matched blood using platelet concentrate from a blood bank ensuring processing under sterile conditions and ruling out infectious diseases. Secondly, al-PRP application to the lower extremity ulcers was local and topical, which may be why there was no immune response, such as an allergic response of the skin. Indeed, animal experiments have been used to investigate the immunogenicity of al-PRP; specifically, an intramuscular injection of al-PRP did not trigger a severe chronic immune response in rabbits [30]. Finally, a possible mechanism of “immune exemption” is the following: al-PRP might be degraded and completely absorbed in the surface of the wound, with little reaching the bloodstream, thereby avoiding most of the alloantigens (human leukocyte antigens and human platelet antigens) [20]. Further, after platelet activation, the structure and levels of expression of platelet surface antigen may be disturbed and reduce immunogenicity [61].

The application of PRP is becoming accepted as a safe and effective treatment for chronic non-healing ulcers and, moreover, it is easy to prepare [47]. While au-PRP has been more widely investigated, the use of allogeneic products has some potential advantages. On the one hand, allogeneic platelets are usually readily available from blood banks, and from this source, they are safe, affordable, and tend to be highly standardised in terms of platelet count, residual white blood cells and red blood cell content. Indeed, the conditions for their preparation are set out in international standards that apply both in the United States and Europe (centrifugation rates for their isolation, centrifugation temperature, separation and processing techniques and composition of the preservative solution) [62]. On the other hand, the majority of patients with diabetic wounds are elderly with long history of anaemia, DM, hypovolaemia, infections, thrombocytopaenia, skin problems, poor nutrition, and immunological dysfunction, and are likely to be on antiplatelet agents [54,63,64] and it is difficult to treat such patients with au-PRP or skin grafts. That is, when the condition of a patient is very poor, it may not be appropriate to draw whole blood to obtain autologous PRP [65], and hence, the interest in using other products including al-PRP. For example, the treatment of neuropathic diabetic foot ulcers through the topical application of platelet releasate (CT-102 APST, activated platelet supernatant, topical; Curative Technologies^®^, Setauket, NY, USA) was found to be successful, with a significant increase in the rate of healing of diabetic foot ulcers [66,67].

Further, there is growing evidence in the literature on the successful use of al-PRP in wound repair from both animal studies [68,69,70] and clinical studies in humans [29,71,72].

To our knowledge, Zhao et al. [31] were the first to report successful treatment of hydroxyurea-related chronic lower extremity ulcers with single-donor al-PRP, in their case, using a donor with lineal consanguinity. They described a patient with ET treated with hydroxyurea whose clinical diagnosis was perplexing [31]. Both hypertension and thrombosis associated with ET and platelet count and blood pressure, which were well controlled during hydroxyurea administration, were ruled out as the main factors underlying the development of ulcers. Finally, she was finally diagnosed with hydroxyurea-related ulcers, consistent with the scientific literature [73], and the ulcers healed completely after al-PRP therapy once a week for 4 weeks.

The success in that case reported could be attributed to various factors. First, al-PRP provides all the functional factors present in au-PRP [74], and as stated above, these include numerous bioactive molecules that have been associated with increased angiogenesis and pain alleviation [75]. Vascular ulcers are often associated with pain, and the authors found that the PRP therapy was extremely effective in alleviating pain in this patient in line with previous research [76]. On the other hand, PRP was used together with a skin substitute and such materials may improve healing in non-healing skin ulcers such as diabetic foot ulcers [77].

Since the case report of Zhao et al. [31], Liao et al. [65] have reported a study assessing the efficacy and safety of al-PRP in the repair of chronic refractory wounds, in a single-centre prospective randomised study including 60 patients (39 men and 21 women, aged 57 ± 10 years old). Participants were divided into al-PRP-treated and control groups, both groups receiving standard care. In this study, al-PRP was obtained by two-step centrifugation of whole blood from healthy individuals and was applied to wounds after debridement. Notably, no cases of allergic reaction or rejection were reported. The authors assessed the clinical effects by a visual examination of the wound and objective evaluation of the wound surface. After 30 days, the al-PRP group showed bright red granulation tissue which bled easily and less inflammatory exudate and the healing rate was much faster in the al-PRP-treated patients than controls. The authors concluded [65] that the treatment of chronic wounds with the combination of standard care and al-PRP significantly reduces healing time.

The study of He et al. [33] published last year is, to our knowledge, the first to compare the feasibility, efficacy and safety of using al-PRP rather than au-PRP in the treatment of CNHWs, potentially overcoming problems associated with the use of the autologous product mentioned above [28].

According to previous studies, PRP concentrations of around 5-fold higher than baseline may be sufficient to obtain adequate results [21,78,79]. He et al. [33] reported the preparation of enriched plasma by separation of autologous or allogenic platelet concentrate, and notably, platelet concentrations obtained did not differ significantly between al-PRP and au-PRP, both reaching levels approximately 6-fold that in whole blood [33]. Notably, we have observed positive outcomes with platelet concentrations of just 2- to 3-fold higher than baseline.

Given these findings, al-PRP from carefully selected donors seems to be a solution that is ready to be used and, compared au-PRP, it avoids the need to draw large volumes of blood from the patient and is associated with less waste of valuable blood resources [80] as well as reducing the risk of poor quality PRP, associated with au-PRP being prepared using variable protocols [28].

### 4.4. PRP Preparation

Importantly, regarding the clinical use of PRP, standards for its preparation and quality assurance have yet to be established and differences in preparation methods can be expected to influence the properties of the product. In relation to the mechanical steps, the use of a higher centrifugation force might yield a higher concentration of platelets, but such high forces might also induce early platelet activation, leading to potential losses of growth factors and in turn efficacy of the PRP [54]. As an alternative to centrifuge methods, Li et al. described a modified procedure for preparing au-PRP gel [81]. In platelet gels, platelets are located within a fibrin network, from where they continuously release bioactive substances and growth factors that disseminate in the surrounding area (Rožman and Bolta, 2007) [82]. Piccin et al. developed a new method seeking to optimise activation and promote gel stability. Specifically, in addition to the usual PRP activation with thrombin and calcium [48], they added coagulation factors I (fibrinogen) and XIII and aprotinin. The first, fibrinogen, is converted to fibrin by thrombin; the second, coagulation factor XIII, promotes the formation of bonds strengthen the fibrin clot, and the third, aprotinin, an antifibrinolytic molecule, inhibits proteolytic enzymes and hence slows down fibrinolysis and increases the consistency and stability of the platelet gel.

In contrast, in our approach, platelets are not activated by calcium chloride or similar agents, but rather a simple freeze-thaw process is used to damage the platelets and prompt the release of granules carrying growth factors, which are what help heal ulcers by promoting granulation and epithelialization. Further, in He et al. [33], preliminary studies by our group [31,36], and the case series reported here, the al-PRP has been prepared with whole blood from a healthy donor using a blood cell separator and can be considered to have been obtained following a standardized protocol.

Notably, the characteristics of platelet products for transfusion are well specified, while the minimum requirements for PRP are less clear [83]. In particular, while there does seem to be a consensus on the minimum number of platelets in the final product [84], less is known about the number of other types of blood cells in PRP that may have an impact on efficacy. White blood cells contain and produce cytokines that are catabolically active and may have an effect on the inflammatory phase of the wound healing process, but which can also have a significant antimicrobial role in PRP [85]. Given that single donor platelets are a standard product in blood banks, residual white blood cell and red blood cell content must be highly standardised [70]. Specifically, the maximum quantity of red and white blood cells should meet the standards of international scientific societies such as the European Committee on Blood Transfusion (2020) [86].

Semenič et al. (2018) [32] sought to ensure a minimum standardisation of the al-PRP they used. Further, the results of extensive laboratory blood tests (complete blood count, electrolytes, urea, creatinine, albumins, proteins, iron, C reactive protein, erythrocyte sedimentation rate, liver function tests, coagulation tests) indicated that neither hydrogel nor allogeneic platelet gel had systemic effects. These authors also [32] updated the formula of precursors and increased the platelet gel stability and concluded that a more complex PRP activation method, using not only a combination of calcium and thrombin but also aprotinin, fibrinogen and coagulation factor XIII, might be more effective. They argued that modern non-adherent dressings absorb less of the more solid platelet gel they used than more liquid forms, and it would therefore be easier to ensure long contact times, and hence, the effects could be expected to last for longer. Nonetheless, covering the recently applied liquid product with a sterile gauze, we have observed successful healing with a much simpler approach.

### 4.5. Implications for Practice

Overall, as well as being safe, al-PRP therapy is painless and patients can be followed-up as outpatients. The simple method we propose, based on highly standardised and affordable allogeneic platelet units readily available through transfusion services, can be considered a feasible treatment option for CNHWs when other approaches have failed. Based on the currently available scientific evidence, we believe that the use of al-PRP in wound healing is effective. If confirmed in future research, it could become the gold standard for the treatment of CNHWs.

Specifically, in our patients, we used PDGF from single-donor apheresis platelets (al-PRP), with a single freeze-thaw process. The PRP preparation protocol involved a single cycle of centrifugation at a rate of 2400–2800 rpm of donor blood obtained using an apheresis machine, and allowed us to obtain a platelet concentration 2- to 3-fold higher than baseline values. While more complex products may have advantages, our simple approach is safe, seems to be effective and is easy-to-implement.

As large enough amounts of al-PRP for use to promote wound healing can be effectively prepared by centrifugation of platelet concentrate, it becomes possible to use PRP for this purpose even in patients who are diabetics and have poor physical and/or mental functioning, and from whom au-PRP or skin grafts may be difficult to obtain. In particular, given the potential red cell damage and white cell loss, plateletpheresis in such patients may put pressure on their already fragile health, in turn exacerbating their disease and delaying wound healing. The need to draw large quantities of blood can be avoided by the use of al-PRP from well-characterised donors, this approach also allowing full use of valuable blood resources.

### 4.6. Limitations

In our study, it was not possible to build a multivariate model due to the small sample size (N = 23). Despite this, we attempted to construct a multiple logistic regression model, including factors with effect sizes of at least 8–10% and removing those making smaller contributions, using a Wald backward procedure. With it approach, we failed to obtain a final model. We then tried using a forward stepwise procedure, introducing one by one the two variables that were most significant in the univariate analysis; however, again, it was not possible to construct a multivariate model, even with these two factors, let alone when including all variables that were close to significance. Therefore, we concluded that we lacked statistical power to obtain a model to explain the factors related to ulcer healing in patients treated with PRP. Further studies with larger sample sizes and long follow-ups are needed to obtain multivariate models to explain which factors favour the healing of ulcers treated with PRP.

## 5. Conclusions

Given the wound healing observed in the patients in our study, the use of PDGF from single donor (apheresis) platelets, with a freeze-thaw process and the PRP preparation protocol described, we could suggest that the use of PRP in the healing of CNHWs is a promising approach. If this were to be confirmed in further research, this method could become the gold standard for the treatment of this type of chronic wound.

## Figures and Tables

**Table 1:** Descriptive analysis of the blood test results.

VARIABLES	Sample (*N* = 23 CNHWs)	
Blood group		
O	56.5% (13)	
A	34.8% (8)	
B	8.7% (2)	
Rh		
Positive	82.6% (19)	
Negative	17.4% (4)	
Irregular antibodies, negative	100% (23)	
Serological results, negative	100% (23)	
Haemoglobin level (g/dL)		
Mean (SD)/Median (Range)	13.90 (1.19)	13.8 (10.1–17.2)
Normal	78.3% (18)	
Elevated	4.3% (1)	
Reduced	17.4% (4)	
White blood cell count (×10^3^ µL)		
Mean (SD)/Median (Range)	9.40 (4.14)	7.6 (4.4–16.2)
Normal	65.2% (15)	
Elevated	34.8% (8)	
Platelet count (×10^3^ µL)		
Mean (SD)/Median (Range)	270 (200)	213 (120–848)
Normal	56.5% (13)	
Elevated	13.0% (3)	
Reduced	30.4% (7)	
Lymphocyte count (×10^3^ µL)		
Mean (SD)/Median (Range)	1.75 (0.59)	1.7 (0.9–2.9)
Normal	74.2% (23)	73.9% (17)
Lymphopaenia	25.8% (8)	26.1% (6)
Cholesterol level (mg/dL)		
Mean (SD)/Median (Range)	156 (37)	154 (108–217)
Normal	65.2% (15)	
Elevated	21.7% (5)	
Reduced	13.0% (3)	
Albumin level (g/dL)		
Mean (SD)/Median (Range)	4.12 (0.37)	4.2 (3.3–4.8)
Normal	95.7% (22)	
Reduced	4.3% (1)	

**Table 2:** Sociodemographic factors associated with wound healing in patients treated with PRP (*N* = 23 CNHWs).

FACTOR	Result of PRP Treatment		Hypothesis Test		Effect Size: R^2^
	Healing (*n* = 13)	No Healing (*n* = 10)	Value	*p* Value	
AGE					
Mean (SD)	71.5 (11.6)	80.3 (4.7)	ZU = 1.85 ^†^	0.064	0.196
Median (Range)	71 (53–86)	79 (77–89)			
Intervals			Chi^2^ = 5.67 ^†^	0.059	0.246
50–69 years	100% (5)	0.0% (–)			
70–79 years	36.4% (4)	63.6% (7)			
89–89 years	57.1% (4)	42.9% (3)			
Sex			Chi^2^ = 3.20 ^†^	0.074	0.139
Men	68.8% (11)	31.3% (5)			
Women	28.6% (2)	71.4% (5)			

^†^ = close to significance.

**Table 3:** Medical history-related factors associated with wound healing in patients treated with PRP (*N* = 23 CNHWs).

FACTOR	Result of PRP Treatment		Hypothesis Test		**Effect Size: R^2^**
	Healing (*n* = 13)	No Healing (*n* = 10)	Value	*p* Value	
Comorbidities			Chi^2^ = 5.79	0.122	0.252
No comorbidities	80.0% (4)	20.0% (1)			
DM + cardiovascular diseases	71.4% (5)	28.6% (2)			
Only cardiovascular diseases	50.0% (4)	50.0% (4)			
Others	0.0% (–)	100% (3)			
Comorbidities			Chi^2^ = 1.43	0.231	0.062
No	80.0% (4)	20.0% (1)			
Yes	50.0% (9)	50.0% (9)			
High blood pressure			Chi^2^ = 0.21	0.645	0.009
Yes	60.0% (9)	40.0% (6)			
No	50.0% (4)	50.0% (4)			
DM			Chi^2^ = 0.91	0.340	0.040
Yes	71.4% (5)	28.6% (2)			
No	50.0% (8)	50.0% (8)			
Wound trigger			Chi^2^ = 2.59	0.274	0.112
No known cause	70.0% (7)	30.0% (3)			
Hydroxyurea	41.7% (5)	58.3% (7)			

DM, diabetes mellitus.

**Table 4:** Blood test result-related factors associated with wound healing in patients treated with PRP (*N* = 23 CNHWs).

FACTOR	Result of PRP Treatment		Hypothesis Test		Effect Size: R^2^
	Healing (*n* = 13)	No Healing (*n* = 10)	Value	*p* Value	
Blood group			Chi^2^ = 2.22	0.329	0.097
O	46.2% (6)	53.8% (7)			
A	62.5% (5)	37.5% (3)			
B	100% (2)	0.0% (–)			
Rh			Chi^2^ = 1.96	0.162	0.085
Positive	63.2% (12)	36.8% (7)			
Negative	25.0% (1)	75.0% (3)			
Haemoglobin level (g/dL)					
Mean (SD)	13.73 (1.55)	14.12 (0.40)	ZU = 1.56	0.118	0.027
Median (Range)	13.6 (10.1–17.2)	14.2 (13.4–14.5)			
Categories			Chi^2^ = 1.64	0.441	0.071
Normal levels	50.0% (9)	50.0% (9)			
Elevated levels	100% (1)	0.0% (–)			
Reduced levels	75.0% (3)	25.0% (1)			
White blood cell count (×10^3^ µL)					
Mean (SD)	7.85 (3.01)	11.41 (4.66)	ZU = 1.97 *	0.049	0.191
Median (Range)	7.2 (4.4–13.6)	14.1 (4.4–16.2)			
Categories			Chi^2^ = 4.96 *	0.026	0.215
Normal levels	73.3% (11)	26.7% (4)			
Elevated	25.0% (2)	75.0% (6)			
Platelet count (×10^3^ µL)					
Mean (SD)	280 (193)	258 (218)	ZU = 0.66	0.513	0.003
Median (Range)	218 (147–848)	213 (120–848)			
Categories			Chi^2^ = 0.79	0.673	0.034
Normal	61.5% (8)	38.5% (5)			
Elevated	66.7% (2)	33.3% (1)			
Reduced	42.9% (3)	57.1% (4)			
Lymphocyte count (×10^3^ µL)					
Mean (SD)	1.52 (0.48)	2.07 (0.61)	ZU = 1.93 ^†^	0.053	0.214
Median (Range)	1.6 (0.9–2.7)	1.7 (1.3–2.9)			
Categories			Chi^2^ = 2.38	0.123	0.103
Normal	47.1% (8)	52.9% (9)			
Lymphopaenia	83.3% (5)	16.7% (1)			
Cholesterol level (mg/dL)					
Mean (SD)	160 (43)	151 (27)	ZU = 0.84	0.401	0.016
Median (Range)	169 (110–217)	154 (108–188)			
Categories			Chi^2^ = 9.44 **	0.009	0.410
Normal	33.3% (5)	66.7% (10)			
Elevated	100% (5)	0.0% (–)			
Reduced	100% (3)	0.0% (–)			
Albumin level (g/dL)					
Mean (SD)	4.13 (0.41)	4.12 (0.34)	ZU = 0.25	0.804	0.000
Median (Range)	4.1 (3.6–4.8)	4.2 (3.3–4.6)			
Categories			Chi^2^ = 1.36	0.244	0.059
Normal	59.1% (13)	40.9% (9)			
Reduced	0.0% (–)	100% (1)			

^†^ = close to significant. * = significant. ** = highly significant.

**Table 5:** Wound-related factors associated with wound healing in patients treated with PRP. (*N* = 23 CNHWs).

FACTOR	Result of PRP Treatment		Hypothesis Test		Effect Size: R^2^
	Healing (*n* = 13)	No Healing (*n* = 10)	Value	*p* Value	
Location of the wound					
Leg + ankle + foot + toes	54.5% (12)	45.5% (10)			
Knee	100% (1)	0.0% (–)			
Size (cm)					
Mean (SD)	3.23 (2.00)	2.90 (1.68)	ZU = 0.34	0.730	0.088
Median (Range)	3.00 (0.5–8.0)	2.50 (1.0–6.5)			
Categories			Chi^2^ = 3.24	0.198	0.141
<2 cm	71.4% (5)	28.6% (2)			
2–3 cm	33.3% (3)	66.7% (6)			
>3 cm	71.4% (2)	28.6% (2)			
Severity			Chi^2^ = 2.35	0.503	0.102
Mild	100% (2)	0.0% (–)			
Moderate	62.5% (5)	37.5% (3)			
Moderate-to-Severe	50.0% (4)	50.0% (4)			
Severe	40.0% (2)	60.0% (3)			
Previous treatments			Chi^2^ = 1.99	0.369	0.087
Only wound care at home	71.4% (5)	28.6% (2)			
Surgery	33.3% (2)	66.7% (4)			
Local wound care at hospital	60.0% (6)	40.0% (4)			
Duration of previous treatment (months)					
Mean (SD)	3.54 (2.50)	2.90 (0.88)	ZU = 0.33	0.974	0.027
Median (Range)	2.0 (1–10)	3.0 (2–4)			
Categories			Chi^2^ = 0.78	0.676	0.034
≤2 months	63.6% (7)	36.4% (4)			
3 months	40.0% (2)	60.0% (3)			
>3 months	57.1% (4)	42.9% (3)			

## Data Availability

Not applicable.
